# Molecular basis of essential and morphological variations across 12 balancer strains in *C. elegans*

**DOI:** 10.17912/micropub.biology.000846

**Published:** 2023-05-26

**Authors:** Filip Cotra, Tatiana Maroilley, Maja Tarailo-Graovac

**Affiliations:** 1 Biochemistry and Molecular Biology, University of Calgary, Calgary, Alberta, Canada

## Abstract

Balancers are primarily chromosomal rearrangements that allow lethal or sterile mutations to be stably maintained as heterozygotes. Strains with balanced lethal/sterile mutations are available at the Caenorhabditis Genetics Center. Such strains harbor morphological markers, with associated molecular changes, that are in *trans* to the balancer. In many cases, only the genetic position (in cM) has been described for balanced mutations or morphological markers. We used short-read whole genome sequencing to uncover the genomic position of those variants (balanced mutations and linked markers) and predicted their effect. We investigated 12 different strains, and characterized at a molecular level 12 variants.

**Figure 1. Molecular basis of balanced mutations and morphological markers across 12 balancer strains:**
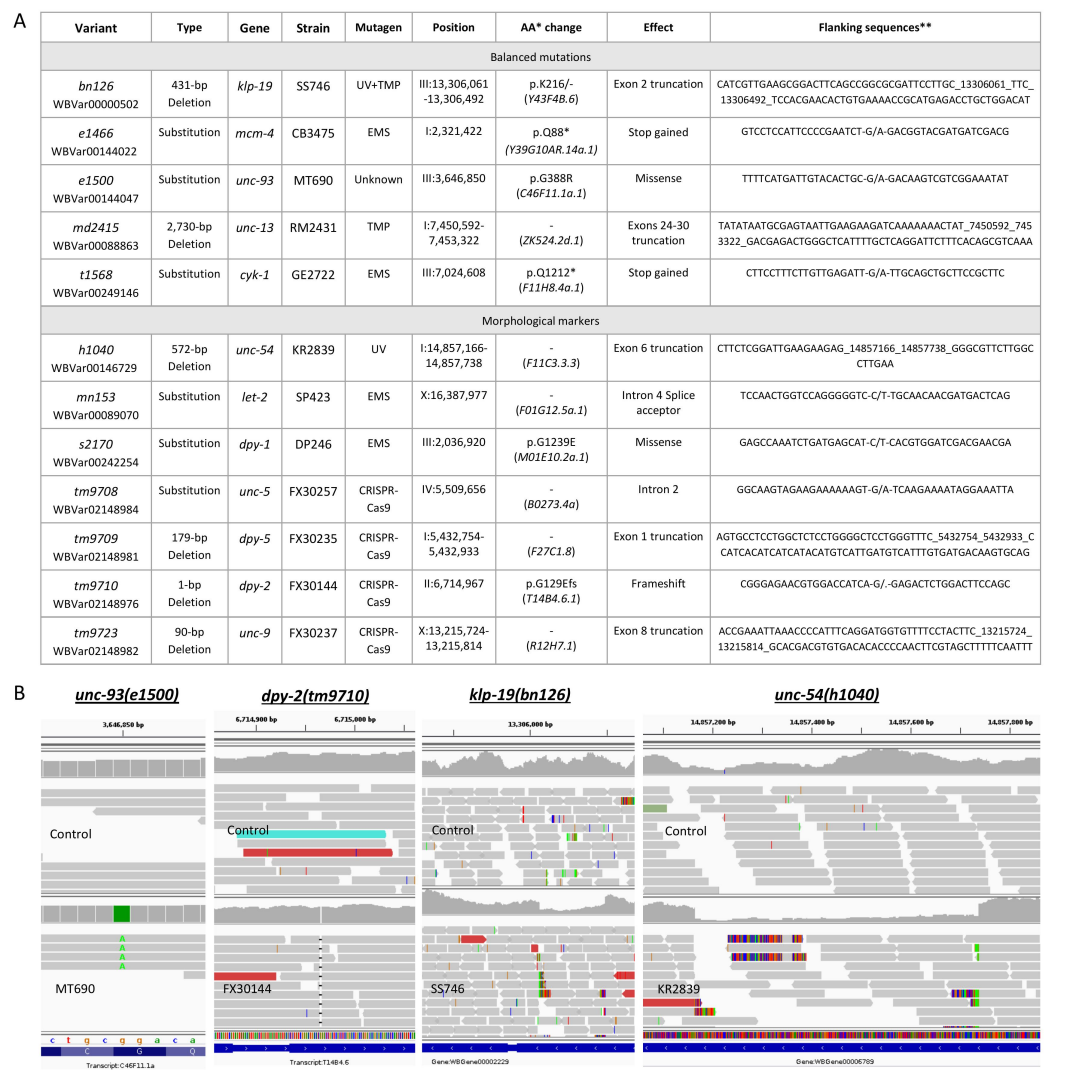
A) Table summarizing balanced mutations (TOP) and morphological markers (BOTTOM) resolved at a molecular level (genomic position and variant effect) in this study. *Amino Acid change. Between parenthesis is the transcript used as reference. For frameshift mutations, we indicated the amino acid after which the frameshift occurs. ** The flanking sequences were retrieved from Sanger sequencing for *bn126* , *md2415* , *tm9709,* and *tm9723* . B) Visualization of the genomic position of four morphological markers or balanced mutations in the Illumina short-read whole genome sequencing data using the Integrative Genomics Viewer v2.16 (IGV) in the control genome ( N2 ) and the strain of interest. For each sample, the information is displayed on two different tracks. The histogram of the top track represents the coverage per base. A sudden drop in the coverage can signal a deletion. The arrows in the bottom track represent the reads aligned along the reference genome. A grey read is a read aligned to the reference genome with limited differences. At a deletion breakpoint, reads colored in red have their mate aligned on the reference genome at a larger distance than the inner distance initially used during the library preparation. The reference sequence and corresponding annotation is displayed at the very bottom of each screenshot. From left to right: *unc-93 ( e1500 )* in MT690 , *dpy-2 (tm9710)* in FX30144 , *klp-19 ( bn126 )* in SS746 , and *unc-54 ( h1040 )* in KR2839 .

## Description

Balancer chromosomes have been used for decades to maintain variations in *Caenorhabditis elegans* ( *C. elegans* ) that impede the organism’s ability to propagate, causing lethality or sterility (Edgley et al., 2006) . Balancers rely on complex genomic rearrangements created through mutagenesis, originally implicating random processes like X-rays or UVs, and more recently via CRISPR-Cas9 (Dejima et al., 2018) . We have recently used short-read whole genome sequencing (srWGS) technology to explore the molecular structure of balancers at base-pair resolution from various different strains available at the Caenorhabditis Genetics Center (CGC). Balancer strains carry balancer chromosomes, balanced detrimental variants and additional distinctive small variants that provide visible markers for either a balancer chromosome or the balanced lethal mutations, called morphological markers (Edgley et al., 2006) . These markers are used to track worms as homozygotes will display a unique and distinctive phenotype (e.g. Uncoordinated-Unc or Dumpy-Dpy) while heterozygotes will present a wild-type phenotype. In addition, balancer strains available at the CGC carry mutations preserved in a heterozygous state on balancer chromosomes, such as the *bn126* variant in the essential gene, *klp-19* (Powers et al., 2004) , that elicit phenotypes like temperature sensitive sterility, developmental defects, or homozygous lethality or sterility. Using our published srWGS datasets (Maroilley et al., 2021, 2022) , we have mapped to base-pair resolution morphological markers and balanced mutations for which the genomic position was so far unknown. Such information is crucial to predict the effect of such variants and better understand the molecular mechanism, as well as the genotype-phenotype relationship.

We previously sequenced balancer strains with paired-end Illumina srWGS to map and explore the structure of genetic balancers (Maroilley et al., 2021, 2022) . Some alleles (balanced mutation and/or markers) reported in the genotype of the strains included in both studies were yet to be mapped at the genomic level. Indeed, while one mutation ( *bn126* ) was known to be a deletion, its genetic position had been interpolated based on phenotypic observations and genetic mapping. We have also included balancer strains created with CRISPR-Cas9 (Dejima et al., 2018) to retrieve the genomic positions for some morphological markers included in those genomes.

Here, we are reporting the genomic location of 12 variants across 12 strains (listed in Figure 1A ). Reads associated with the genes affected by each variation were manually and visually examined to identify their potential location. As a proof of concept, four of these variations (deletions *bn126* , *md2415* , *tm9709* , and *tm9723* ) were confirmed through PCR and Sanger sequencing.

As morphological markers and balanced mutations have been described affecting specific genes to create their unique morphological phenotype or lethality/sterility, we visually analysed the sequence of those genes within their respective strains, in comparison to the genome of N2 which we sequenced and used as a control ( Figure 1B ). Often, only one variant could be detected in the gene of interest, absent in the N2 genome. This made identification unambiguous, as each variation was associated with only one possible originating mutation. Of these variants, six were base substitutions while six were deletions (including one 1-bp deletion). *bn126* was identified as a deletion of 431 bp through position III:13,306,061-13,306,492 ( Figure 1B ). It causes the truncation of exon 2 of *klp-19* . *e1466* is a G>A transition at position I:2,321,422 ( Figure 1B ), resulting in a nonsense mutation affecting a glutamine (Q/*). *e1500* was identified as a missense mutation causing a G>A transition at position III:3,646,850, thus changing glycine to arginine (G/R). *h1040* was found to be a deletion of 572 bp, through position I:14,857,166-14,857,738 ( Figure 1B ), truncating exon 6 of *unc-54* . *md2415* was also identified as a deletion, removing 2,730 bp through position I:7,450,592-7,453,322, truncating partially exon 24 and exon 30, and entirely exons 25 to 29 of *unc-13* . *mn153* is a C>T transition at position X:16,387,977, falling within an intronic region, and thus causing no amino acid change, but predicted to affect splicing (splice acceptor variant). *s2170* was identified as a C>T transition at position III:2,036,920, acting as a missense mutation by changing glycine to valine (G/E). *t1568* was found to be a G>A transition at position III:7,024,608, resulting in the creation of a stop codon. *tm9708* was revealed to be a G>A transition at position IV:5,509,656 and is an intronic variant. The prediction of its effect would require further functional analyses (RNA-Seq). *tm9709* was found to be a deletion of 179 bp through position I:5,432,754-5,432,933, truncating partially exon 1 of *dpy-5* . *tm9710* was identified as a single base deletion at position II:6,714,967, removing a guanine and causing a frameshift ( Figure 1B ). Finally, *tm9723* was found to be a deletion of 90 base pairs through position X:13,215,724-13,215,814, truncating partially exon 8 of *unc-9* .

Using PCR and Sanger sequencing, we experimentally validated the genomic positions retrieved from the srWGS analysis for four deletions: *klp-19* ( *bn126* ) in SS746 , *md2415* ( *unc-13* ) in RM2431 , *dpy-5* ( *tm9709* ) in FX30235 and *unc-9* ( *tm9723* ) in FX30237 . Primers used are described in Methods and Sanger sequences can be found in Figure 1A . Deletions analysed in this study were found to have been submitted to mutagenesis processes prone to DNA breakage such as UV/TMP and Psoralen (that intercalates in DNA and under UVA exposure, can create covalent interstrand cross-links). The substitutions, however, were predominantly found in strains exposed to EMS. These observations agree with previous studies (Gengyo-Ando & Mitani, 2000) .

Mapping morphological markers and lethal/sterile mutations preserved on balancer chromosomes to the base-pair resolution may give greater insight into how they impact the genes they affect and the phenotype or survival of their associated strains. It also offers the opportunity to genotype strains at molecular level, to complement phenotyping. It has the potential to uncover important gene regions for biochemical pathways and support the documentation of variation, expanding opportunities for future research while solidifying prior efforts.

## Methods

**Obtaining Strains and maintenance**

All strains used in this analysis were provided by the CGC, which is funded by the NIH Office of Research Infrastructure Programs (P40 0D010440). All strains were maintained at 16˚C and kept on standard NGM plates streaked with OP50 .

**Whole Genome Sequencing**

Genomic DNA was collected and extracted as described in Maroilley et al. (2022). In brief, the DNA was extracted using Qiagen blood and tissue kit (13323) from ~100 mg of worm tissue and diluted with 10 nM Tris-HCl (pH 8.0). We sequenced the genomes with PCR-free library preparation protocol and NovaSeq 600 Illumina sequencing technology.

**Read Alignment and Analysis**

FastQC v0.11.9 (Andrews S., 2010) was used to analyze the sequence quality of the fastq files obtained from sequencing, Trimmomatic v0.39 (Bolger et al., 2014) was used to trim adaptors from the end of Illumina reads, and Burrows-Wheeler Aligner (BWA v0.7.17) (Li, 2013) was used to align the trimmed sequences using the Maximal Exact Match (MEM) algorithm.

**Identification of Variations**

Sequences were analyzed using Integrative Genomics Viewer v2.16 (IGV) to identify potential sources for each variation (Robinson et al., 2011) , making use of the WS245 *C. elegans* reference to identify regions associated with the genes of interest. Along with the strains thought to contain the variations, N2 was observed in a similar manner to filter out any mutations contained within, as these surely could not be the cause of the observed phenotypic impact associated with the variations. The effect of variants was predicted using Variant Effect Predictor Ensembl Release 109 (McLaren et al., 2016) .

**Experimental Validation**

We confirmed four of the deletions by PCR and sanger sequencing. The primers used are as follows: for *bn126* , 5’-TTGTCCACCAAGTGCCGTTTC-3’ and 5’-CTCGTCAGTGCGTTGTTCCAAAG-3’; for *md2415* 5’-TCCTTAAGACACCCTCCTACA-3’ and 5’-AGTCCTTTGCCACCTGTAAA-3’; for *tm9709* 5’-CGAGACCTGCCATCGTATTT-3’ and 5’-CGAGACCTGCCATCGTATTT-3’; for *tm9723* 5’-GTAGTTAGAACTTTCCCGGTTCA-3’ and 5’-AAACCACAGAAGGAAGGAGAAG-3’.

## Reagents

**Table:** 

**STRAIN**	**GENOTYPE**	**AVAILABLE FROM**
SS746	*klp-19 ( bn126 )/mT1 [ dpy-10 ( e128 )] III. *	CGC
CB3475	*mcm-4 ( e1466 )/szT1 [ lon-2 ( e678 )] I; +/szT1 X. *	CGC
MT690	* nDf6/ unc-93 ( e1500 ) dpy-17 ( e164 ) III. *	CGC
KR2839	*hDf15 unc-75 ( e950 )/hIn1 [ unc-54 ( h1040 )] I. *	CGC
RM2431	*unc-13 ( md2415 )/hT1 I; +/hT1 V. *	CGC
SP423	*mnDp1 (X;V)/+ V; unc-3 ( e151 ) let-2 ( mn153 ) X. *	CGC
GE2722	*cyk-1 ( t1568 ) unc-32 ( e189 )/qC1 [ dpy-19 ( e1259 ) glp-1 ( q339 )] III; him-3 ( e1147 ) IV. *	CGC
DP246	*unc-45 ( st601 )/sC1 [ dpy-1 ( s2170 )] III. *	CGC
FX30257	* tmC25 [ unc-5 (tm9708)] IV. *	CGC
FX30235	* tmC20 [ dpy-5 (tm9709)] I. *	CGC
FX30144	* tmC6 [ dpy-2 (tm9710)] II. *	CGC
FX30237	* tmC24 [ unc-9 (tm9723)] X. *	CGC
